# Potential Roles of Selectins in Periodontal Diseases and Associated Systemic Diseases: Could They Be Targets for Immunotherapy?

**DOI:** 10.3390/ijms232214280

**Published:** 2022-11-18

**Authors:** Mei Zhong, Jiangyong Huang, Zhe Wu, Kok-Gan Chan, Lijing Wang, Jiang Li, Learn-Han Lee, Jodi Woan-Fei Law

**Affiliations:** 1 Novel Bacteria and Drug Discovery Research Group (NBDD), Microbiome and Bioresource Research Strength (MBRS), Jeffrey Cheah School of Medicine and Health Sciences, Monash University Malaysia, Bandar Sunway 47500, Selangor Darul Ehsan, Malaysia; 2 Department of Prosthodontics, Affiliated Stomatology Hospital of Guangzhou Medical University, Guangzhou 510180, China; 3 Guangzhou Key Laboratory of Basic and Applied Research of Oral Regenerative Medicine, Guangdong Engineering Research Center of Oral Restoration and Reconstruction, Affiliated Stomatology Hospital of Guangzhou Medical University, Guangzhou 510180, China; 4 Division of Genetics and Molecular Biology, Institute of Biological Sciences, Faculty of Science, University of Malaya, Kuala Lumpur 50603, Malaysia; 5 International Genome Centre, Jiangsu University, Zhenjiang 212013, China; 6 Vascular Biology Research Institute, School of Life Sciences and Biopharmaceutics, Guangdong Pharmaceutical University, Guangzhou 510006, China

**Keywords:** periodontal disease, selectin, systemic disease, diabetes, cardiovascular disease, host immune response, therapeutic target

## Abstract

Periodontal diseases are predisposing factors to the development of many systemic disorders, which is often initiated via leukocyte infiltration and vascular inflammation. These diseases could significantly affect human health and quality of life. Hence, it is vital to explore effective therapies to prevent disease progression. Periodontitis, which is characterized by gingival bleeding, disruption of the gingival capillary’s integrity, and irreversible destruction of the periodontal supporting bone, appears to be caused by overexpression of selectins in periodontal tissues. Selectins (P-, L-, and E-selectins) are vital members of adhesion molecules regulating inflammatory and immune responses. They are mainly located in platelets, leukocytes, and endothelial cells. Furthermore, selectins are involved in the immunopathogenesis of vascular inflammatory diseases, such as cardiovascular disease, diabetes, cancers, and so on, by mediating leukocyte recruitment, platelet activation, and alteration of endothelial barrier permeability. Therefore, selectins could be new immunotherapeutic targets for periodontal disorders and their associated systemic diseases since they play a crucial role in immune regulation and endothelium dysfunction. However, the research on selectins and their association with periodontal and systemic diseases remains limited. This review aims to discuss the critical roles of selectins in periodontitis and associated systemic disorders and highlights the potential of selectins as therapeutic targets.

## 1. Introduction

Periodontal diseases (PD), such as gingivitis and periodontitis, are a global public health problem with a prevalence up to 50% of the reported oral health cases [1]. The growing global perception is that periodontal health improvements will contribute to better health [2]. Periodontal diseases comprise inflammatory pathologies and microbiome dysbiotic events in the host that lead to tooth loss, edentulism, and occlusal dysfunction. Additionally, they can trigger several chronic systemic diseases [3], negatively impacting an individual’s health and well-being [4]. During periodontal infections, periodontal pathogens may play a crucial role in driving leukocytes to extravasate into the whole circulatory system through the ulcerated and inflamed surface of the gingival [5]. The vascular endothelium permeability is generally influenced by pathogen-associated molecules, host-derived proinflammatory cytokines, and adhesion molecules, which cause the barrier to disintegrate. These events then initiate the inflammatory process through mediating firm adhesion and transmigration of leukocytes [6,7]. The capture and rolling of leukocytes on platelets and vascular endothelium are essential for the onset of inflammation [8].

Selectins are an important component of adhesion molecules, and they are cell membrane glycoproteins expressed on leukocytes, platelets, and endothelial cells in the bloodstream that mediate host immune response [9,10]. There are three selectin subfamily members: Leukocyte-selectin (L-selectin, CD62L), Platelet-selectin (P-selectin, CD62P), and Endothelial-selectin (E-selectin, CD62E) [10]. Their primary ligand (P-selectin glycoprotein ligand-1(PSGL-1, SELPLG)) on the surface of leukocytes interacts with P-, L-, and E-selectin, each with varying affinity to mediate adhesion and rolling of leukocytes on endothelium, and other signal transduction in the immune response to inflammation [11]. The selectin family has been well-studied as an adhesion molecule in inflammatory diseases [12].

In recent years, selectins and their ligands have been discovered as one of the important factors connecting periodontal inflammation and the development of other systemic disorders, including chronic inflammatory diseases such as cardiovascular diseases (CVD) [13], diabetes [14], atherosclerosis [15], certain cancers, and others [16,17]. The expressions of selectins and PSGL-1 could vary according to the severity of periodontitis. Furthermore, clinical evidence has indicated that, after reducing the adhesion of periodontal pathogens in periodontal diseases by various non-surgical and surgical treatments, systemic inflammation is subsequently reduced with changes in circulating levels of selectins [18,19]. Therefore, selectins could be a potential novel therapeutic target for the prevention or symptom alleviation of periodontal and associated systemic diseases. However, it is essential to acknowledge that the research on selectins’ roles and regulatory mechanisms in periodontal diseases, and related systemic inflammation is still limited.

Hence, this review aims to shed light on the current immune mechanisms of selectins and their biological role and function in periodontal diseases and associated systemic diseases, especially looking into endothelial function, leukocyte adhesion, and immune regulation. In order to collect the information for this review, the literature search was conducted using three databases (OVID Medicine, Scopus, Pubmed). The terms ‘periodontitis’ OR ‘periodontal disease’ OR ‘periodontal pathogen’ AND ‘selectin’ AND ‘systemic disease’ OR ‘systemic disorder’ OR ‘diabetes’ OR ‘cardiovascular disease’ were used in the search. Thirty-nine studies, including animal studies and clinical trials, were selected for qualitative analysis based on their relevance to the main content of this review.

## 2. The Functions and Biological Role of Selectins

Selectins are a crucial component of adhesion molecules [20,21]. They are cell transmembrane glycoproteins composed of a C-type lectin domain, an epidermal growth factor (EGF)-like domain, and extracellular domains of short cytoplasmic tail [22], which effectively mediate cell-to-cell adhesion by recognizing the cell surface’s carbohydrates [23,24]. These adhesion processes are pivotal in chronic and acute inflammatory diseases, infection, cancer, homing of bone marrow stem cells and lymphocytes, and immune cell surveillance [12]. The selectin family consists of L-selectin, P-selectin, and E-selectin, each of which has distinct roles and cell adhesion characteristics [22]. P-selectin is selectively expressed on activated platelets, which contributes to the adhesion of platelets to leukocytes, such as neutrophils, monocytes, and natural killer (NK) cells, driving immune cells to the inflammatory sites [25,26]. P-selectin and E-selectin are typically located on endothelial cells that trigger proinflammatory cytokines like tumor necrosis factor (TNF)-α, interleukin -1β (IL-1β), and so forth [27]. L-selectin is predominantly expressed on naive T- and B cells, myeloid cells, and leukocytes, which can mediate the recirculation of lymphoid cells and leukocyte adhesion [28]. Additionally, PSGL-1, a homodimeric type I mucin-like transmembrane protein with disulfide bonds, has been identified as a principal common ligand binding P-, L-, and E-selectin, and it can be expressed on platelets, neutrophils, monocytes, and most peripheral B cells and T cells [29]. PSGL-1, interacting with endothelial P- and E-selectin, can mediate the initial interactions of tethering and roll of leukocytes. Subsequently, it binds L-selectin to increase recruiting leukocytes at the inflammation site [30,31,32].

Previous studies have revealed the significant role of selectins and PSGL-1 in many normal physiological functions. Nonetheless, studies also have reported on a series of important adhesion processes that occur in several inflammatory diseases and cancers [10,33,34]. As a result, elevated levels of selectins often participate in the process of human diseases, including cardiovascular diseases (CVD) [13], psoriasis [35,36], kidney disease [37], asthma, chronic obstructive pulmonary disease (COPD) [38], thrombosis [39], arthritis [40], and cancer disease [16,17]. P- and E-selectins are found on activated endothelial cells, mediating leukocyte homing and trafficking [41,42,43,44,45]. Therefore, they may serve as a biomarker for endothelial activation in CVD (e.g., coronary heart disease, atherosclerosis, hypercholesterolemia, and hypertension) [46,47,48,49]. It is noteworthy that upregulated P- and E-selectin expression has been observed in gingival crevicular fluid and peripheral blood of psoriasis, rheumatoid arthritis, and many other inflammatory and infective diseases [12,50,51,52,53].

In addition, selectins may contribute to various aspects of tumor progression and migration [54,55,56]. Studies have reported the functions of selectins in the interaction of activated platelets, leukocytes, and endothelial cells with cancer cells [57,58], thus promoting the spread of cancer cells in the blood flow by exploiting the tethering and rolling adhesion cascade of leukocytes [16,17,59]. Selectins and their ligands are potent mediators of leukocyte–endothelium interaction under inflammatory conditions since they facilitate leukocyte rolling and extravasation at inflammation sites of the endothelium in postcapillary venules and platelet activation of inflammatory periodontal tissues [60,61,62]. Hence, the overexpression of selectins and secretion of proinflammatory cytokines are important factors initiating inflammatory responses.

## 3. The Role of Selectins in Periodontal Diseases and Associated Systemic Diseases

Periodontitis is a chronic inflammatory illness that progressively deteriorates the integrity of the supporting periodontal tissues due to the subgingival biofilm matrix on the tooth surface [63]. Disruption of periodontal pocket epithelial integrity usually facilitates the entry of bacteria, bacterial products, and cytokines into the bloodstream, resulting in bacteremia and systemic inflammation [64,65]. Periodontal diseases are closely associated with systemic diseases through leukocyte accumulation caused by periodontal bacteria, resulting in endothelial dysfunction and causing inflammation of the distal organs [66,67,68]. Generally, the systemic inflammatory state caused by severe periodontitis is characterized by the release of proinflammatory cytokines, leukocytosis, and changes in the status of endothelial cells and platelets in the circulatory system. These events further exacerbate periodontitis via a positive feedback mechanism [69,70].

Selectins and their ligands are located in essential sites where the leukocyte performs functions associated with platelet activation and endothelial dysfunction. Thus, their overexpression could trigger inflammatory and cell-mediated host immunological responses [71,72,73,74]. Several studies have demonstrated the high expression levels of E-selectin and P-selectin on gingival endothelial cells of patients with adult periodontitis. The severity of periodontal damage is also correlated to high levels of TNF-α and IL-1 found in sites of gingival inflammation [75,76,77]. On the contrary, total P/E-selectin deficiency may not be beneficial, as it could increase the susceptibility to early-onset periodontal disease caused by infection. Niederman et al. created engineered P/E-selectin-deficient mice (P/E^−/−^) to test this hypothesis, and the results of the study demonstrated that a significantly earlier onset of alveolar bone loss was observed in P/E^−/−^ mice than that in the wild-type mice [78].

Henceforth, this section will provide an overview of the potential mechanisms in the connection between periodontitis and several common systemic diseases, followed by insights into the selectins’ expression and biological function in association with periodontitis and systemic diseases.

### 3.1. Periodontitis–Diabetes Linkage

**Figure 1:**
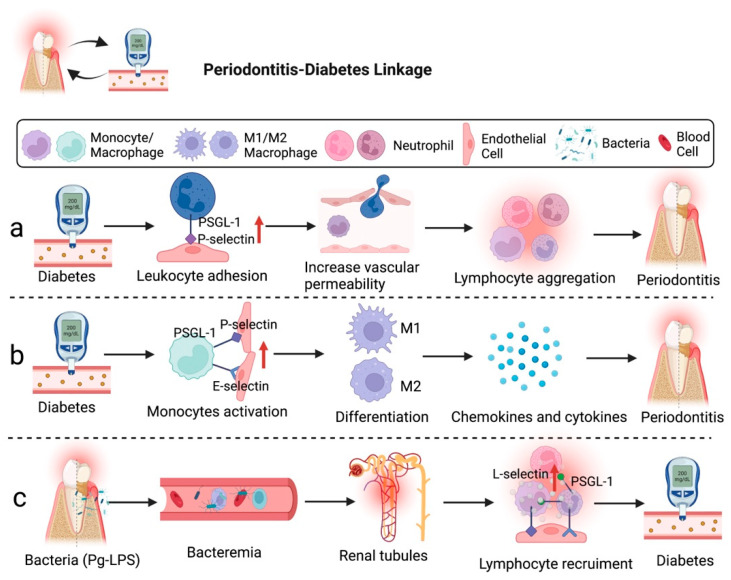
Diabetes can accelerate the development of periodontitis through the involvement of selectins. (**a**) Hyperglycemia can increase vascular permeability and leukocyte adhesion molecule expression, which enhance leukocyte aggregation and lead to periodontitis. (**b**) Blood-derived macrophages/monocytes secrete chemokines and cytokines to regulate the development of diabetes-associated periodontitis. (**c**) Bacteremia caused by Pg-LPS may induce chronic renal inflammation. Figure created with BioRender.com.

The relationship between diabetes and chronic periodontal disease is characterized by an enhanced local to systemic inflammatory response. Bacteria and host immune responses are two main factors affecting the severity and progression of periodontal tissue destruction [79,80,81]. Several studies have reported that patients with type 2 diabetes (t2DM) had a high prevalence of periodontal disease in the periodontitis–diabetes linkage. The increased expression of selectins and other inflammatory mediators was frequently associated with higher severity of periodontal conditions due to host tissue destruction, which could be a characteristic of periodontitis-t2DM (Figure 1) [14,82].

Research demonstrated that chronic hyperglycemia caused by diabetes creates a microvascular environment similar to acute inflammation in the gingiva with periodontitis. The chronic hyperglycemia state is induced by overexpression of P-selectin in endothelial cells and PSGL-1 in leukocytes, leading to the alteration of the permeability of gingival postcapillary venules and resulting in lymphocyte spillage [83]. An animal study on leukocyte activation revealed that the cytoplasmic domain of PSGL-1 is dispensable as it is not required to support leukocyte rolling on P-selectin. However, the cytoplasmic domain of PSGL-1 is still necessary to activate lymphocyte function-associated antigen 1 (LFA1) to slow rolling on intercellular adhesion molecule-1 (ICAM-1). Hence, the increase in the interaction of P-selectin and PSGL-1 may affect the endothelial cell cytoskeleton, directly affecting vascular permeability [84]. In addition, Lalla et al. emphasized the potential mechanism for the production of tumor necrosis factor-α (TNF-α) by macrophage-derived foam cells macrophages/monocytes, which is related to the activation of E-selectin to mediate the development of diabetes-associated periodontitis [85].

Likewise, severe periodontitis may lead to bacteremia because microorganisms from the oral cavity could reach the renal circulation of individuals with periodontitis-associated diabetes via systemic circulation (Figure 1). It appears that lipopolysaccharides (LPS) of *P. gingivalis* (Pg-LPS) accumulated in glomeruli may cause chronic renal inflammation and damage renal tubules as an impact of the leukocyte migration. Alternatively, the severe stage of diabetes can be complicated by nephropathy through induced diabetic renal inflammation by Pg-LPS, such as glomerulosclerosis and tubulitis. Glomerular overexpression of VCAM-1 and E-selectin induces the recruitment of Mac-1/podoplanin-positive macrophages, leading to the deposition of unmetabolized angiotensin-converting enzyme 2 (ACE2) and fibroblast growth factor 23 (FGF23) and inflammation-induced kidney injury in diabetics [86]. Therefore, periodontal disease may influence patients’ different stages of diabetic progression.

### 3.2. Periodontitis–Cardiovascular Disease Linkage

**Figure 2:**
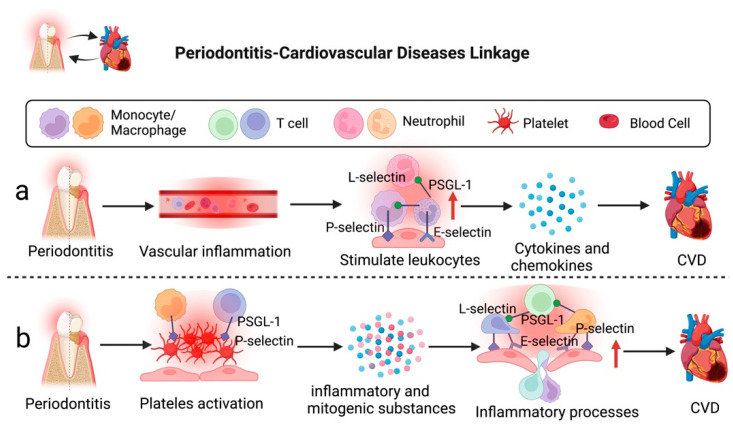
Severe periodontitis can accelerate the development of CVD via selectins. (**a**) Severe inflammation in the periodontal environment can elevate vascular inflammation, leading to the release of proinflammatory cytokines and chemokines, which could result in CVD. (**b**) Activated platelets interact with endothelium to accelerate inflammatory processes and enhance immune cell recruitment, leading to the development of CVD. Figure created with BioRender.com.

Millions of people are afflicted with CVD, causing prolonged suffering, economic loss, and even death. Atherosclerosis is the primary cause of CVD, such as myocardial infarction, heart failure, and claudication [87]. Epidemiological findings have revealed that individuals with severe periodontitis have a higher risk for CVD [88,89,90]. The cause of periodontitis-associated CVD is mainly due to elevated vascular inflammation, which is affected by soluble-intercellular adhesion molecules and immune complexes. This condition induces proinflammatory cytokines and chemokines production, which subsequently stimulate the release of leukocytes, such as neutrophils, monocytes/macrophages, NK cells, and T cells [91]. Moreover, platelet count and activity elevation are other factors for periodontitis and CVD [92]. Activated platelets release potent inflammatory and mitogenic chemicals into the immediate surroundings that affect the endothelium’s chemotactic and adhesive characteristics [93,94]. These molecules work in combination to accelerate inflammatory processes and increase the recruitment of immune cells, resulting in endothelial activation and proliferation [92]. Endothelial activation and adhesion molecule expression appear in atherosclerosis’s initial phase, allowing mononuclear leukocytes to bind to the endothelium and penetrate the intima [95]. Therefore, the development of periodontitis-associated CVD could be promoted by leukocyte activation and recruitment via interaction between adhesion molecules and endothelial cells, in addition to other contributing factors relating to glucose metabolism, lipid metabolism, amino acid metabolism, and phospholipid metabolism [96,97]. Figure 2 illustrates the potential roles of selectins in periodontitis-associated CVD.

Regarding the roles of selectins and their ligands in periodontitis-CVD linkage, Ning et al. conducted a study to investigate the molecular mechanisms between atherosclerosis and periodontitis. Bioinformatics results of the study revealed that Selectin P Ligand (SELPLG) is one of the core crosstalk genes involved in the mechanisms between atherosclerosis and periodontitis. SELPLG was identified to promote the progression of atherosclerosis via regulating the interaction of activated immune cells and endothelial cells and also elevating expression levels of TNF-α and IL6 [15]. Nonetheless, it remains inconclusive whether periodontitis-triggered systemic inflammation can cause the upregulation of SELPLG [15,98]. Additionally, Leong et al. reported that periodontitis-CVD was associated with the elevated expression of serum P-selectin (sP-selectin) level and platelet activation as observed via spider-form and pathological aggregation patterns of platelets [99]. The activated P-selectin could also assist platelets in adhering to monocytes by integrating with PSGL-1, indirectly causing inflammation and pathological changes of periodontitis related to thromboembolism [100,101].

### 3.3. Periodontitis and Other Diseases Linkage

Other periodontitis-related diseases, such as cancer (periodontitis-cancer), are also worth exploring. Recent studies discovered that PSGL-1 could act as an immune checkpoint and therapeutic target for tumor disease. PSGL-1 binds on T cells and potentially contributes to inhibitory signaling pathways that promote T cell exhaustion in tumors; thus, blocking PSGL-1 can increase tumor control [102]. At the tumor site, several adhesion molecules (such as E- and P-selectins) may act in a synergistic way to control endothelial progenitor cell (EPC) integration and tumor angiogenesis [103]. Moreover, animal studies showed that a protein from *P. gingivalis* accelerated tumor growth by increasing E-selectin expression and enhancing EPC’s function [104,105].

**Figure 3:**
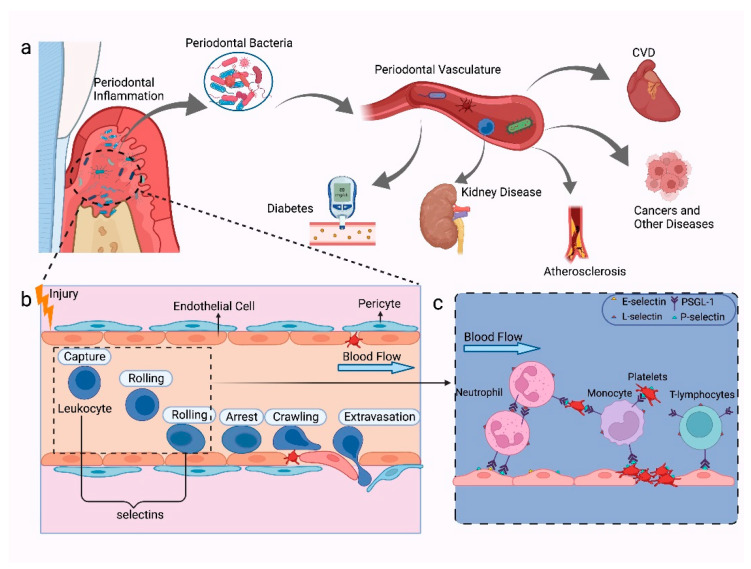
The mechanisms of selectins in periodontal diseases and systemic diseases. (**a**) Periodontal diseases caused by subgingival bacteria on the tooth surface and periodontal pocket. Periodontal bacteria can enter the bloodstream and cause systemic inflammation due to disruption in the integrity of the vascular endothelium, such as CVD, diabetes, kidney diseases, atherosclerosis, and certain cancers. (**b**) In a severe inflammation state, the host’s immune response will be triggered, which causes the activation of various lymphocytes in the vasculature, rolling of leukocytes on the vascular endothelium, spillover, and activation of platelets. (**c**) P-selectin is selectively expressed on activated platelets which connect platelet adhesion and leukocytes, such as neutrophils, monocytes, and T-lymphocytes; E-selectin and P-selectin are generally expressed on endothelial cells that activate the process of rolling of leukocytes; L-selectin is mainly expressed on leukocytes, which can mediate the recirculation of lymphoid cells and leukocytes adhesion; PSGL-1 can interact with P-, L-, and E-selectin, which can be expressed on platelets, neutrophils, monocytes, and most peripheral T cells and B cells. Figure created with BioRender.com.

Overall, only a few studies focus on the role of selectins in periodontitis and associated systemic disease. Figure 3 illustrates the potential functions of selectins in periodontal diseases and systemic diseases. Further research is crucial to examine the mechanisms of selectins in these systemic diseases, and clinical trials to evaluate their prospects as immunotherapeutic targets are required.

### 3.4. The Relationship between Selectins and Periodontal Pathogens

Pathogens’ perturbation of the host’s oral microbiome can disrupt gingival health. During the events of subgingival infections, the inflammatory immune response produced is the primary contributor to periodontitis. The three most common bacterial species associated with periodontitis are *Aggregatibacter actinomycetemcomitans*, *Porphyromonas gingivalis*, and *Tannerella forsythia* [106]. Bacteremia induced by periodontal bacteria is common during biting and tooth brushing [64]. The link between periodontitis and systemic disorders stems from the hematogenous dissemination of periodontal pathogens or spillover of inflammatory mediators from periodontal tissues to the whole bloodstream [107].

Several studies have focused on investigating the expression of selectins with periodontal pathogens to understand further the mechanisms between periodontitis and diabetes, CVD, and other systemic diseases [77,107,108]. According to Horie et al., *T. forsythia* in the periodontal pocket could trigger the onset of periodontitis by binding to lectins expressed on host cells, including the E-, P-, and L-selectins, Sig-lec-5, -9, and -10, and DC-SIGN [109]. *T. forsythia* and *A. actinomycetemcomitans* have been detected in human atherosclerotic plaques, which supported the direct link of the periodontal pocket to systemic circulation [110,111]. A study also reported that spontaneously hyperlipidemic mice with atherosclerosis infected by *A. actinomycetemcomitans* presented an increased expression of adhesion molecule (e.g., ICAM-1, E-selectin, and P-selectin) [112]. Assinger et al. revealed that periodontal pathogens, *A. actinomycetemcomitans* and *P. gingivalis*, induced rapid surface expression of P-selectin in platelets and endothelial cells. This significantly increased plasma soluble P-selectin (sP-selectin) levels, correlated with the severity of periodontitis and bacterial infection [76]. sP-selectin has the ability to promote the severity of thrombotic and CVD via stimulating leukocyte recruitment to vascular injury and escalating CVD progression [62]. Nicu et al. also found that platelets from periodontitis patients had increased exposure of P-selectin and formation of platelet monocyte complexes compared to controls. The enhanced platelet activation was in response to periodontal pathogens. Activated platelets and leukocytes are risk factors triggering atherothrombotic activity, increasing the risk for CVD [77].

Besides, it has been discovered that human aortic endothelial cells (HAEC) infected with invasive *P. gingivalis* can lead to the expression of E-/P-selectin, ICAM-1, and VCAM-1, thereby enhancing the expression of tissue factor and producing procoagulant effect [113,114]. Additionally, studies reported that *P. gingivalis*, *A. actinomycetemcomitans*, and other dental plaque microbes, such as *Streptococcus sanguis,* become a potential risk for cerebrovascular and atherosclerotic disorders by inducing platelet activation and aggregation. This causes a high release rate of P-selectin [115,116]. Furthermore, *P. gingivalis* can trigger endothelial cells and stimulate E-selectin overexpression, which enhances monocytes’ adherence to endothelial cells [117,118] and initiates vascular inflammation [119]. Lipopolysaccharides (LPS) present on the surface membrane of periodontal pathogen *P. gingivalis* can cause periodontal tissue destruction, a risk factor for systemic diseases [120]. LPS of *P. gingivalis* (Pg-LPS) is widely recognized by host defense systems via Toll-like receptor 4 (TLR4) or Toll-like receptor 2 (TLR2) to activate immune response [121,122]. Kajiwara et al. reported that LPS from *P. gingivalis* caused diabetic renal inflammation, which includes glomerulosclerosis and tubulitis. Overexpression of E-selectin was observed in renal intertubular capillaries, parenchyma, and glomeruli, which led to macrophage infiltration and kidney damaged by Pg-LPS-induced inflammation in diabetes [86].

Additionally, other experiments showed that NOD1 and NOD2, as classical nucleotide-binding oligomerization domain (NOD)-like receptors, play an essential role in sensing intracellular pathogens [123]. *P. gingivalis* has been demonstrated to activate NOD1, NOD2, and TLR2 expression in human endothelial cells, leading to enhanced E-selectin expression. In *P. gingivalis*-regulated endothelial cells, selectins are implicated in the activation of the NF-κB signaling pathway. Meanwhile, additional signaling pathways through P38 MAPK are primarily presented during NOD1 activation in response to *P. gingivalis* infection [108,124,125]. Promoting the recruitment of monocytes and T cells and their subsequent adherence to the endothelium could accelerate the first stage of atherogenesis [126]. In contrast, another important immunodominant antigen in individuals with periodontitis is GroEL from *P. gingivalis*, which may be involved in pathological processes and systemic inflammation [127]. Lin et al., 2015 conducted an animal study to understand the underlying mechanisms between oral bacteria and cancer. They revealed that the GroEL protein of *P. gingivalis* increased E-selectin expression and promoted neovascularization via PI3K- and p38 MAPK-signaling pathways and partially via a NOS-related pathway [105].

Overall, periodontal pathogens destroy periodontal tissues and induce inflammation of distal organs through their capacity to release various virulence factors, such as LPS and GroEL, to cause platelet activation, endothelium dysfunction, and lymphocyte infiltration [105,128]. Table 1 summarizes the potential mechanisms in the association between periodontal pathogens, selectins, and periodontitis-associated systemic diseases. However, the research investigating the relationship between periodontal bacteria and selectins in periodontitis-associated diseases is limited, and the findings remain inconclusive.

## 4. The Implication of Selectins in Treatment of PD and Associated Systemic Diseases

Endothelial dysfunction forms the basis for developing periodontal and systemic inflammatory diseases. The upregulation of adhesion molecules in periodontitis can increase circulating levels of systemic inflammation. Therefore, selectively reducing the cell adhesion molecules on endothelium that facilitate the uptake of leukocytes into the vessel wall is a promising method to limit the development of periodontitis and may aid in controlling related systemic diseases [129]. Periodontal therapy, which mainly consists of the mechanical destruction of the subgingival calculus and microbial film of the diseased teeth, is often associated with a local and systemic repairment of endothelial dysfunction [71,73,129,130].

Various non-surgical and surgical options are available to treat periodontal diseases by reducing the adhesion of periodontal pathogens. These have been proven to represent a novel therapy in decreasing the risk of vascular disease [18,130]. Clinical non-surgical periodontal treatment could reduce systemic inflammation by decreasing inflammatory mediators’ circulating levels, such as CRP, sE-selectin, IL-1, IL-6, and TNF-α [19,131]. Non-surgical therapy of periodontitis-associated diabetic individuals can significantly reduce the serum levels of high-sensitivity-C-reactive protein (hsCRP) and E-selectin. Additionally, an in vivo study indicated that biomarkers associated with vascular function (E-Selectin and si-CAM plasma levels) were significantly reduced in the obesity–periodontitis rats after local combination with systemic therapy [132]. However, another study showed an interesting result where sP-selectin levels had increased after three months of periodontal therapy [133]. The increased circulating sP-selectin could be derived from membrane-bound P-selectin rapidly shedding from activated platelets after periodontal therapy [133,134], while platelets can still maintain their normal function and characteristics. In addition, the shed sP-selectin may have a “calming” effect on activating neutrophils and mediating leukocyte adhesion [135], which may be part of a healing phase after treatment. The study found that sP-selectin levels in periodontitis patients were much lower than that of untreated periodontitis and healthy controls from previous studies [92,133,136]. The inconsistencies in sP-selectin levels observed across different studies could be due to the employment of different anticoagulants for the analysis (e.g., citrate plasma or EDTA plasma).

There are a limited number of clinical trials involving selectins as a therapeutic target. Nevertheless, studies have proven that periodontal therapy positively influences endothelial-dependent function by altering selectins’ expression. Given the potential roles of selectin in periodontitis and associated systemic diseases, selectins can be anticipated to represent possible novel therapeutic targets for the treatment of these diseases.

## 5. Conclusions

Periodontal diseases are among the highest prevalent public health problem worldwide. Periodontal diseases are closely associated with systemic inflammatory disorders, especially the development of diabetes and cardiovascular disease [124]. People suffering from periodontitis and systemic diseases have a greater social-economic burden and lower quality of life. Periodontal inflammation causes a systemic inflammatory immune response. The inflammatory factors in leukocytes, platelets, and endothelium, as well as the direct lesion of the intima by bacteria in the circulation, could induce the formation and development of inflammatory debris [137]. During this process, the selectins and their ligands play vital roles in mediating the rolling and transfer of leukocytes in blood flow and promoting the development of other inflammatory disorders [138]. The regulation of selectins’ levels could offer preventative measures in the course of periodontal diseases and their associated inflammatory disorders. Therefore, selectins may be a promising therapeutic target for periodontal diseases and periodontal-associated systemic disorders. Thus far, the limited knowledge of selectins’ functions and regulatory mechanisms in periodontal diseases and their associated systemic diseases warrants further investigation. Further research in these areas is required to assist in developing selectin-based therapeutic strategies for the treatment of periodontal and systemic diseases.

## Figures and Tables

**Table 1:** The roles of periodontal pathogens and selectins involved in periodontitis associated-systemic diseases.

Periodontal Pathogens	Selectin	Systemic Disease	Main Role of Selectin	Reference
*P. gingivalis*	P-selectin /PSGL-1	CVD; Atherosclerosis	Induce platelet activation and aggregation	[115,116]
	E-selectin	CVD	Facilitate monocytes adhering to endothelial cells	[117,118]
			LPS is recognized Via TLR4 and TLR2 to mediate the function of endothelium	[121,122]
			Facilitate monocytes and T cells recruiting and adhering to endothelium	[126]
			Promote neovascularization through PI3K- and p38 MAPK-signaling pathways, as well as a NOS-related pathway	[105]
		Atherosclerosis	Activate NOD1, NOD2, and TLR2 expression to regulate the function of endothelium	[108,124,125]
		Diabetes	Induce diabetic renal inflammation by infiltrating Mac-1-positive macrophages	[86]
*A. actinomycetemcomitans*	P-,E-selectin /PSGL-1	Atherosclerosis; CVD	Induce platelet activation and aggregation data	[115,116]
			Regulate macrophage and T cells through cytokine response	[111]
*T. forsythia*	E-,P-,L- selectin	Atherosclerosis	Modulate the host immune response through regulating macrophage and T cells	[109,111]

## Data Availability

Not applicable.

## References

[1] Chen M.X., Zhong Y.J., Dong Q.Q., Wong H.M., Wen Y.F. (2021). Global, regional, and national burden of severe periodontitis, 1990–2019: An analysis of the Global Burden of Disease Study 2019. J. Clin. Periodontol..

[2] Tonetti M.S., Jepsen S., Jin L., Otomo-Corgel J. (2017). Impact of the global burden of periodontal diseases on health, nutrition and wellbeing of mankind: A call for global action. J. Clin. Periodontol..

[3] Hajishengallis G. (2015). Periodontitis: From microbial immune subversion to systemic inflammation. Nat. Rev. Immunol..

[4] Reynolds I., Duane B. (2018). Periodontal disease has an impact on patients’ quality of life. Evid. -Based Dent..

[5] Räisänen I.T., Umeizudike K.A., Pärnänen P., Heikkilä P., Tervahartiala T., Nwhator S.O., Grigoriadis A., Sakellari D., Sorsa T. (2020). Periodontal disease and targeted prevention using aMMP-8 point-of-care oral fluid analytics in the COVID-19 era. Med. Hypotheses.

[6] Ley K., Laudanna C., Cybulsky M.I., Nourshargh S. (2007). Getting to the site of inflammation: The leukocyte adhesion cascade updated. Nat. Rev. Immunol..

[7] Folwaczny M., Karnesi E., Berger T., Paschos E. (2017). Clinical association between chronic periodontitis and the leukocyte extravasation inhibitors developmental endothelial locus-1 and pentraxin-3. Eur. J. Oral Sci..

[8] Lam F.W., Brown C.A., Valladolid C., Emebo D.C., Palzkill T.G., Cruz M.A. (2020). The vimentin rod domain blocks P-selectin-P-selectin glycoprotein ligand 1 interactions to attenuate leukocyte adhesion to inflamed endothelium. PLoS ONE.

[9] Zarbock A., Ley K., McEver R.P., Hidalgo A. (2011). Leukocyte ligands for endothelial selectins: Specialized glycoconjugates that mediate rolling and signaling under flow. Blood J. Am. Soc. Hematol..

[10] Ley K. (2001). Functions of selectins. Mamm. Carbohydr. Recognit. Syst..

[11] Tinoco R., Otero D.C., Takahashi A.A., Bradley L.M. (2017). PSGL-1: A new player in the immune checkpoint landscape. Trends Immunol..

[12] Ley K. (2003). The role of selectins in inflammation and disease. Trends Mol. Med..

[13] Berezin A.E. (2015). Impaired phenotype of circulating endothelial-derived microparticles: Novel marker of cardiovascular risk. J. Cardiol. Ther..

[14] Geisinger M.L., Michalowicz B.S., Hou W., Schoenfeld E., Gelato M., Engebretson S.P., Reddy M.S., Hyman L. (2016). Systemic inflammatory biomarkers and their association with periodontal and diabetes-related factors in the diabetes and periodontal therapy trial, a randomized controlled trial. J. Periodontol..

[15] Ning W., Ma Y., Li S., Wang X., Pan H., Wei C., Zhang S., Bai D., Liu X., Deng Y. (2021). Shared molecular mechanisms between atherosclerosis and periodontitis by analyzing the transcriptomic alterations of peripheral blood monocytes. Comput. Math. Methods Med..

[16] Bendas G., Borsig L. (2012). Cancer cell adhesion and metastasis: Selectins, integrins, and the inhibitory potential of heparins. Int. J. Cell Biol..

[17] Natoni A., Macauley M.S., O’Dwyer M.E. (2016). Targeting selectins and their ligands in cancer. Front. Oncol..

[18] Fischer R.G., Lira Junior R., Retamal-Valdes B., Figueiredo L.C.d., Malheiros Z., Stewart B., Feres M. (2020). Periodontal disease and its impact on general health in Latin America. Section V: Treatment of periodontitis. Braz. Oral Res..

[19] El-Shinnawi U., Soory M. (2013). Associations between periodontitis and systemic inflammatory diseases: Response to treatment. Recent Pat. Endocr. Metab. Immune Drug Discov..

[20] Harjunpää H., Llort Asens M., Guenther C., Fagerholm S.C. (2019). Cell adhesion molecules and their roles and regulation in the immune and tumor microenvironment. Front. Immunol..

[21] Ley K. (2007). Adhesion Molecules: Function and Inhibition.

[22] McEver R.P., Zhu C. (2010). Rolling cell adhesion. Annu. Rev. Cell Dev. Biol..

[23] Feizi T. (2001). Carbohydrate ligands for the leukocyte-endothelium adhesion molecules, selectins. Mamm. Carbohydr. Recognit. Syst..

[24] Taylor M.E., Drickamer K. (2007). Paradigms for glycan-binding receptors in cell adhesion. Curr. Opin. Cell Biol..

[25] Bonfanti R., Furie B.C., Furie B., Wagner D.D. (1989). PADGEM (GMP140) is a component of Weibel-Palade bodies of human endothelial cells. Blood.

[26] Chacko A.-M., Hood E.D., Zern B.J., Muzykantov V.R. (2011). Targeted nanocarriers for imaging and therapy of vascular inflammation. Curr. Opin. Colloid Interface Sci..

[27] Tedder T.F., Steeber D.A., Chen A., Engel P. (1995). The selecting: Vascular adhesion molecules. FASEB J..

[28] Rosen S.D. (2004). Ligands for L-selectin: Homing, inflammation, and beyond. Annu. Rev. Immunol..

[29] Ratcliffe M.J. (2016). Encyclopedia of Immunobiology.

[30] Ley K., Bullard D.C., Arbonés M.L., Bosse R., Vestweber D., Tedder T.F., Beaudet A.L. (1995). Sequential contribution of L-and P-selectin to leukocyte rolling in vivo. J. Exp. Med..

[31] Jung U., Bullard D.C., Tedder T.F., Ley K. (1996). Velocity differences between L-and P-selectin-dependent neutrophil rolling in venules of mouse cremaster muscle in vivo. Am. J. Physiol. -Heart Circ. Physiol..

[32] Ley K., Tedder T.F., Kansas G.S. (1993). L-selectin can mediate leukocyte rolling in untreated mesenteric venules in vivo independent of E-or P-selectin. Blood.

[33] Ley K., Kansas G.S. (2004). Selectins in T-cell recruitment to non-lymphoid tissues and sites of inflammation. Nat. Rev. Immunol..

[34] McEver R.P. (2015). Selectins: Initiators of leucocyte adhesion and signalling at the vascular wall. Cardiovasc. Res..

[35] Bock D., Philipp S., Wolff G. (2006). Therapeutic potential of selectin antagonists in psoriasis. Expert Opin. Investig. Drugs.

[36] Schon M.P., Drewniok C., Boehncke W. (2004). Targeting selectin functions in the therapy of psoriasis. Curr. Drug Targets-Inflamm. Allergy.

[37] Rabelink T.J., De Boer H.C., Van Zonneveld A.J. (2010). Endothelial activation and circulating markers of endothelial activation in kidney disease. Nat. Rev. Nephrol..

[38] Romano S., Slee D. (2001). Targeting selectins for the treatment of respiratory diseases. Curr. Opin. Investig. Drugs (Lond. Engl. 2000).

[39] Merten M., Thiagarajan P. (2004). P-selectin in arterial thrombosis. Z. Für Kardiol..

[40] Sfikakis P., Mavrikakis M. (1999). Adhesion and lymphocyte costimulatory molecules in systemic rheumatic diseases. Clin. Rheumatol..

[41] Su Y., Lei X., Wu L., Liu L. (2012). The role of endothelial cell adhesion molecules P-selectin, E-selectin and intercellular adhesion molecule-1 in leucocyte recruitment induced by exogenous methylglyoxal. Immunology.

[42] Subramaniam M., Saffaripour S., Van De Water L., Frenette P.S., Mayadas T.N., Hynes R.O., Wagner D.D. (1997). Role of endothelial selectins in wound repair. Am. J. Pathol..

[43] Matsumoto M., Atarashi K., Umemoto E., Furukawa Y., Shigeta A., Miyasaka M., Hirata T. (2005). CD43 functions as a ligand for E-Selectin on activated T cells. J. Immunol..

[44] Zhang J., DeFelice A.F., Hanig J.P., Colatsky T. (2010). Biomarkers of endothelial cell activation serve as potential surrogate markers for drug-induced vascular injury. Toxicol. Pathol..

[45] Zhang J., Hanig J.P., De Felice A.F. (2012). Biomarkers of endothelial cell activation: Candidate markers for drug-induced vasculitis in patients or drug-induced vascular injury in animals. Vasc. Pharmacol..

[46] Hwang S.-J., Ballantyne C.M., Sharrett A.R., Smith L.C., Davis C.E., Gotto Jr A.M., Boerwinkle E. (1997). Circulating adhesion molecules VCAM-1, ICAM-1, and E-selectin in carotid atherosclerosis and incident coronary heart disease cases: The Atherosclerosis Risk In Communities (ARIC) study. Circulation.

[47] Rathouska J., Jezkova K., Nemeckova I., Nachtigal P. (2015). Soluble endoglin, hypercholesterolemia and endothelial dysfunction. Atherosclerosis.

[48] Perkins L.A., Anderson C.J., Novelli E.M. (2019). Targeting P-selectin adhesion molecule in molecular imaging: P-selectin expression as a valuable imaging biomarker of inflammation in cardiovascular disease. J. Nucl. Med..

[49] Chironi G., Dosquet C., Del-Pino M., Denarie N., Megnien J.-L., Drouet L., Bal dit Sollier C., Levenson J., Simon A. (2006). Relationship of circulating biomarkers of inflammation and hemostasis with preclinical atherosclerotic burden in nonsmoking hypercholesterolemic men. Am. J. Hypertens..

[50] Silva M., Videira P.A., Sackstein R. (2018). E-selectin ligands in the human mononuclear phagocyte system: Implications for infection, inflammation, and immunotherapy. Front. Immunol..

[51] Ludwig R.J., Schön M.P., Boehncke W.-H. (2007). P-selectin: A common therapeutic target for cardiovascular disorders, inflammation and tumour metastasis. Expert Opin. Ther. Targets.

[52] Ishikawa H., Nishibayashi Y., Kita K., Ohno O., Imura S., Hirata S. (1993). Adhesion molecules in the lymphoid cell distribution in rheumatoid synovial membrane. Bull. (Hosp. Jt. Dis. (New York NY)).

[53] Valenzuela F., Fernández J., Jiménez C., Cavagnola D., Mancilla J.F., Astorga J., Hernández M., Fernández A. (2021). Identification of IL-18 and Soluble Cell Adhesion Molecules in the Gingival Crevicular Fluid as Novel Biomarkers of Psoriasis. Life.

[54] Hill C.A.S. (2011). Interactions between endothelial selectins and cancer cells regulate metastasis. Front. Biosci..

[55] Läubli H., Borsig L. (2010). Selectins promote tumor metastasis. Seminars in Cancer Biology.

[56] Konstantopoulos K., Thomas S.N. (2009). Cancer cells in transit: The vascular interactions of tumor cells. Annu. Rev. Biomed. Eng..

[57] Chen M., Geng J.-G. (2006). P-selectin mediates adhesion of leukocytes, platelets, and cancer cells in inflammation, thrombosis, and cancer growth and metastasis. Arch. Immunol. Et Ther. Exp..

[58] Kim Y.J., Borsig L., Han H.-L., Varki N.M., Varki A. (1999). Distinct selectin ligands on colon carcinoma mucins can mediate pathological interactions among platelets, leukocytes, and endothelium. Am. J. Pathol..

[59] Cagnoni A.J., Perez Saez J.M., Rabinovich G.A., Mariño K.V. (2016). Turning-off signaling by siglecs, selectins, and galectins: Chemical inhibition of glycan-dependent interactions in cancer. Front. Oncol..

[60] Zheng F., Chevalier J., Zhang L., Virgil D., Ye S., Kwiterovich P. (2001). An HphI polymorphism in the E-selectin gene is associated with premature coronary artery disease. Clin. Genet..

[61] Houshmand B., Rafiei A., Hajilooi M., Mani-Kashani K., Gholami L. (2009). E-selectin and L-selectin polymorphisms in patients with periodontitis. J. Periodontal Res..

[62] Woollard K., Kling D., Kulkarni S., Dart A.M., Jackson S., Chin-Dusting J. (2006). Raised plasma soluble P-selectin in peripheral arterial occlusive disease enhances leukocyte adhesion. Circul. Res..

[63] Lockhart P.B., Brennan M.T., Sasser H.C., Fox P.C., Paster B.J., Bahrani-Mougeot F.K. (2008). Bacteremia associated with toothbrushing and dental extraction. Circulation.

[64] Forner L., Larsen T., Kilian M., Holmstrup P. (2006). Incidence of bacteremia after chewing, tooth brushing and scaling in individuals with periodontal inflammation. J. Clin. Periodontol..

[65] Li X., Kolltveit K.M., Tronstad L., Olsen I. (2000). Systemic diseases caused by oral infection. Clin. Microbiol. Rev..

[66] Ivetic A., Hoskins Green H.L., Hart S.J. (2019). L-selectin: A major regulator of leukocyte adhesion, migration and signaling. Front. Immunol..

[67] Bui F.Q., Almeida-da-Silva C.L.C., Huynh B., Trinh A., Liu J., Woodward J., Asadi H., Ojcius D.M. (2019). Association between periodontal pathogens and systemic disease. Biomed. J..

[68] Gurav A.N. (2014). The implication of periodontitis in vascular endothelial dysfunction. Eur. J. Clin. Investig..

[69] Loos B.G., Craandijk J., Hoek F.J., Dillen P.M.W.v., Van Der Velden U. (2000). Elevation of systemic markers related to cardiovascular diseases in the peripheral blood of periodontitis patients. J. Periodontol..

[70] Khumaedi A.I., Purnamasari D., Wijaya I.P., Soeroso Y. (2019). The relationship of diabetes, periodontitis and cardiovascular disease. Diabetes Metab. Syndr. Clin. Res. Rev..

[71] Tonetti M.S., D’Aiuto F., Nibali L., Donald A., Storry C., Parkar M., Suvan J., Hingorani A.D., Vallance P., Deanfield J. (2007). Treatment of periodontitis and endothelial function. N. Engl. J. Med..

[72] Mendes R.T., Fernandes D. (2016). Endothelial dysfunction and periodontitis: The role of inflammatory serum biomarkers. Dent. Hypotheses.

[73] Higashi Y., Goto C., Jitsuiki D., Umemura T., Nishioka K., Hidaka T., Takemoto H., Nakamura S., Soga J., Chayama K. (2008). Periodontal infection is associated with endothelial dysfunction in healthy subjects and hypertensive patients. Hypertension.

[74] Yang Q., Langston J.C., Tang Y., Prabhakarpandian B., Kilpatrick L.E., Kiani M.F. (2021). A Microphysiological System to Study Leukocyte-Endothelial Cell Interaction during Inflammation. J. Vis. Exp. Jove.

[75] Velickovic M., Arsenijevic A., Acovic A., Arsenijevic D., Milovanovic J., Dimitrijevic J., Todorovic Z., Milovanovic M., Kanjevac T., Arsenijevic N. (2021). Galectin-3, possible role in pathogenesis of periodontal diseases and potential therapeutic target. Front. Pharmacol..

[76] Assinger A., Buchberger E., Laky M., Esfandeyari A., Brostjan C., Volf I. (2011). Periodontopathogens induce soluble P-selectin release by endothelial cells and platelets. Thromb. Res..

[77] Nicu E., Van der Velden U., Nieuwland R., Everts V., Loos B. (2009). Elevated platelet and leukocyte response to oral bacteria in periodontitis. J. Thromb. Haemost..

[78] Niederman R., Westernoff T., Lee C., Mark L., Kawashima N., Ullman-Culler M., Dewhirst F., Paster B., Wagner D., Mayadas T. (2001). Infection-mediated early-onset periodontal disease in P/E-selectin-deficient mice. J. Clin. Periodontol..

[79] Van Dyke T.E. (2009). Guest Editorial: The Etiology and Pathogenesis of Periodontitis Revisited.

[80] Southerland J.H., Taylor G.W., Moss K., Beck J.D., Offenbacher S. (2006). Commonality in chronic inflammatory diseases: Periodontitis, diabetes, and coronary artery disease. Periodontol. 2000.

[81] Haffajee A.D., Socransky S.S. (2005). Microbiology of periodontal diseases: Introduction. Periodontol. 2000.

[82] Graves D.T., Ding Z., Yang Y. (2020). The impact of diabetes on periodontal diseases. Periodontol. 2000.

[83] Sima C., Rhourida K., Van Dyke T., Gyurko R. (2010). Type 1 diabetes predisposes to enhanced gingival leukocyte margination and macromolecule extravasation in vivo. J. Periodontal Res..

[84] Miner J.J., Xia L., Yago T., Kappelmayer J., Liu Z., Klopocki A.G., Shao B., McDaniel J.M., Setiadi H., Schmidtke D.W. (2008). Separable requirements for cytoplasmic domain of PSGL-1 in leukocyte rolling and signaling under flow. Blood J. Am. Soc. Hematol..

[85] Lalla E., Kaplan S., Yang J., Roth G., Papapanou P., Greenberg S. (2007). Effects of periodontal therapy on serum C-reactive protein, sE-selectin, and tumor necrosis factor-α secretion by peripheral blood-derived macrophages in diabetes. A pilot study. J. Periodontal Res..

[86] Kajiwara K., Sawa Y., Fujita T., Tamaoki S. (2021). Immunohistochemical study for the expression of leukocyte adhesion molecules, and FGF23 and ACE2 in P. gingivalis LPS-induced diabetic nephropathy. BMC Nephrol..

[87] Frostegård J. (2013). Immunity, atherosclerosis and cardiovascular disease. BMC Med..

[88] Sanz M., Del Castillo A.M., Jepsen S., Gonzalez-Juanatey J.R., D’Aiuto F., Bouchard P., Chapple I., Dietrich T., Gotsman I., Graziani F. (2020). Periodontitis and cardiovascular diseases: Consensus report. J. Clin. Periodontol..

[89] Suzuki J.-i., Aoyama N., Ogawa M., Hirata Y., Izumi Y., Nagai R., Isobe M. (2010). Periodontitis and cardiovascular diseases. Expert Opin. Ther. Targets.

[90] Izumi Y., Nagasawa T., Umeda M., Kobayashi H., Takeuchi Y., Yashiro R., Hormdee D., Suda T., Ushida Y., Wara-aswapati N. (2009). Periodontitis and cardiovascular diseases: The link and relevant mechanisms. Jpn. Dent. Sci. Rev..

[91] Tew J., El Shikh M., El Sayed R., Schenkein H. (2012). Dendritic cells, antibodies reactive with oxLDL, and inflammation. J. Dent. Res..

[92] Papapanagiotou D., Nicu E.A., Bizzarro S., Gerdes V.E., Meijers J.C., Nieuwland R., van der Velden U., Loos B.G. (2009). Periodontitis is associated with platelet activation. Atherosclerosis.

[93] Huo Y., Ley K.F. (2004). Role of platelets in the development of atherosclerosis. Trends Cardiovasc. Med..

[94] Lindemann S., Krämer B., Seizer P., Gawaz M. (2007). Platelets, inflammation and atherosclerosis. J. Thromb. Haemost..

[95] Campbell K.A., Lipinski M.J., Doran A.C., Skaflen M.D., Fuster V., McNamara C.A. (2012). Lymphocytes and the adventitial immune response in atherosclerosis. Circul. Res..

[96] Li B., Lu X., Wang J., He X., Gu Q., Wang L., Yang Y. (2018). The metabonomics study of P-selectin glycoprotein ligand-1 (PSGL-1) deficiency inhibiting the progression of atherosclerosis in LDLR-/-mice. Int. J. Biol. Sci..

[97] Luo W., Wang H., Öhman M.K., Guo C., Shi K., Wang J., Eitzman D.T. (2012). P-selectin glycoprotein ligand-1 deficiency leads to cytokine resistance and protection against atherosclerosis in apolipoprotein E deficient mice. Atherosclerosis.

[98] Wang X.-l., Deng H.-f., Tan C.-y., Xiao Z.-h., Liu M.-d., Liu K., Zhang H.-l., Xiao X.-z. (2019). The role of PSGL-1 in pathogenesis of systemic inflammatory response and coagulopathy in endotoxemic mice. Thromb. Res..

[99] Leong X.-F., Ng C.-Y., Badiah B., Das S. (2014). Association between hypertension and periodontitis: Possible mechanisms. Sci. World J..

[100] da Costa Martins P., García-Vallejo J.-J.s., Van Thienen J.V., Fernandez-Borja M., Van Gils J.M., Beckers C., Horrevoets A.J., Hordijk P.L., Zwaginga J.-J. (2007). P-selectin glycoprotein ligand-1 is expressed on endothelial cells and mediates monocyte adhesion to activated endothelium. Atertio. Thromb. Vasc. Biol..

[101] Yu K.-m., Inoue Y., Umeda M., Terasaki H., Chen Z.-y., Iwai T. (2011). The peridontal anaerobe Porphyromonas gingivalis induced platelet activation and increased aggregation in whole blood by rat model. Thromb. Res..

[102] Tinoco R., Carrette F., Barraza M.L., Otero D.C., Magaña J., Bosenberg M.W., Swain S.L., Bradley L.M. (2016). PSGL-1 is an immune checkpoint regulator that promotes T cell exhaustion. Immunity.

[103] Sonsuwan N., Suchachaisri S., Chaloeykitti L. (2011). The relationships between cephalometric parameters and severity of obstructive sleep apnea. Auris Nasus Larynx.

[104] Maeda H., Miyamoto M., Kokeguchi S., Kono T., Nishimura F., Takashiba S., Murayama Y. (2000). Epitope mapping of heat shock protein 60 (GroEL) from Porphyromonas gingivalis. FEMS Immunol. Med. Microbiol..

[105] Lin F.Y., Huang C.Y., Lu H.Y., Shih C.M., Tsao N.W., Shyue S.K., Lin C.Y., Chang Y.J., Tsai C.S., Lin Y.W. (2015). The Gro EL protein of Porphyromonas gingivalis accelerates tumor growth by enhancing endothelial progenitor cell function and neovascularization. Mol. Oral Microbiol..

[106] Van Winkelhoff A., Loos B., Van Der Reijden W., Van Der Velden U. (2002). Porphyromonas gingivalis, Bacteroides forsythus and other putative periodontal pathogens in subjects with and without periodontal destruction. J. Clin. Periodontol..

[107] Teles R., Wang C.Y. (2011). Mechanisms involved in the association between peridontal diseases and cardiovascular disease. Oral Dis..

[108] Wan M., Liu J., Wu D., Chi X., Ouyang X. (2015). E-selectin expression induced by P orphyromonas gingivalis in human endothelial cells via nucleotide-binding oligomerization domain-like receptors and Toll-like receptors. Mol. Oral Microbiol..

[109] Horie T., Inomata M., Into T., Hasegawa Y., Kitai N., Yoshimura F., Murakami Y. (2016). Identification of OmpA-like protein of Tannerella forsythia as an O-linked glycoprotein and its binding capability to lectins. PLoS ONE.

[110] Jia R., Hashizume-Takizawa T., Du Y., Yamamoto M., Kurita-Ochiai T. (2015). Aggregatibacter actinomycetemcomitans induces Th17 cells in atherosclerotic lesions. Pathog. Dis..

[111] Håheim L.L., Schwarze P.E., Thelle D.S., Nafstad P., Rønningen K.S., Olsen I. (2020). Low levels of antibodies for the oral bacterium Tannerella forsythia predict cardiovascular disease mortality in men with myocardial infarction: A prospective cohort study. Med. Hypotheses.

[112] Zhang T., Kurita-Ochiai T., Hashizume T., Du Y., Oguchi S., Yamamoto M. (2010). Aggregatibacter actinomycetemcomitans accelerates atherosclerosis with an increase in atherogenic factors in spontaneously hyperlipidemic mice. FEMS Immunol. Med. Microbiol..

[113] Chou H.-H., Yumoto H., Davey M., Takahashi Y., Miyamoto T., Gibson III F.C., Genco C.A. (2005). Porphyromonas gingivalis fimbria-dependent activation of inflammatory genes in human aortic endothelial cells. Infect. Immun..

[114] Roth G., Moser B., Huang S., Brandt J., Huang Y., Papapanou P., Schmidt A., Lalla E. (2006). Infection with a periodontal pathogen induces procoagulant effects in human aortic endothelial cells. J. Thromb. Haemost..

[115] Lourbakos A., Yuan Y., Jenkins A.L., Travis J., Andrade-Gordon P., Santulli R., Potempa J., Pike R.N. (2001). Activation of protease-activated receptors by gingipains from Porphyromonas gingivalis leads to platelet aggregation: A new trait in microbial pathogenicity. Blood J. Am. Soc. Hematol..

[116] Wu T., Trevisan M., Genco R.J., Dorn J.P., Falkner K.L., Sempos C.T. (2000). Periodontal disease and risk of cerebrovascular disease: The first national health and nutrition examination survey and its follow-up study. Arch. Intern. Med..

[117] Roth G.A., Moser B., Roth-Walter F., Giacona M.B., Harja E., Papapanou P.N., Schmidt A.M., Lalla E. (2007). Infection with a periodontal pathogen increases mononuclear cell adhesion to human aortic endothelial cells. Atherosclerosis.

[118] Hashizume T., Kurita-Ochiai T., Yamamoto M. (2011). Porphyromonas gingivalis stimulates monocyte adhesion to human umbilical vein endothelial cells. FEMS Immunol. Med. Microbiol..

[119] Komatsu T., Nagano K., Sugiura S., Hagiwara M., Tanigawa N., Abiko Y., Yoshimura F., Furuichi Y., Matsushita K. (2012). E-selectin mediates Porphyromonas gingivalis adherence to human endothelial cells. Infect. Immun..

[120] Liccardo D., Cannavo A., Spagnuolo G., Ferrara N., Cittadini A., Rengo C., Rengo G. (2019). Periodontal disease: A risk factor for diabetes and cardiovascular disease. Int. J. Mol. Sci..

[121] Berezow A.B., Ernst R.K., Coats S.R., Braham P.H., Karimi-Naser L.M., Darveau R.P. (2009). The structurally similar, penta-acylated lipopolysaccharides of Porphyromonas gingivalis and Bacteroides elicit strikingly different innate immune responses. Microb. Pathog..

[122] Coats S.R., Berezow A.B., To T.T., Jain S., Bainbridge B.W., Banani K.P., Darveau R.P. (2011). The lipid A phosphate position determines differential host Toll-like receptor 4 responses to phylogenetically related symbiotic and pathogenic bacteria. Infect. Immun..

[123] Opitz B., Eitel J., Meixenberger K., Suttorp N. (2009). Role of Toll-like receptors, NOD-like receptors and RIG-I-like receptors in endothelial cells and systemic infections. Thromb. Haemost..

[124] Walter C., Zahlten J., Schmeck B., Schaudinn C., Hippenstiel S., Frisch E., Hocke A.C., Pischon N., Kuramitsu H.K., Bernimoulin J.-P. (2004). Porphyromonas gingivalis strain-dependent activation of human endothelial cells. Infect. Immun..

[125] Zhang D., Zheng H., Zhao J., Lin L., Li C., Liu J., Pan Y. (2011). Porphorymonas gingivalis induces intracellular adhesion molecule-1 expression in endothelial cells through the nuclear factor-kappaB pathway, but not through the p38 MAPK pathway. J. Periodontal Res..

[126] Andrukhov O., Steiner I., Liu S., Bantleon H.P., Moritz A., Rausch-Fan X. (2015). Different effects of Porphyromonas gingivalis lipopolysaccharide and TLR2 agonist Pam3CSK4 on the adhesion molecules expression in endothelial cells. Odontology.

[127] Ueki K., Tabeta K., Yoshie H., Yamazaki K. (2002). Self-heat shock protein 60 induces tumour necrosis factor-α in monocyte-derived macrophage: Possible role in chronic inflammatory periodontal disease. Clin. Exp. Immunol..

[128] Chung S.W., Kang H.S., Park H.R., Kim S.J., Kim S.J., Choi J.I. (2003). Immune responses to heat shock protein in Porphyromonas gingivalis-infected periodontitis and atherosclerosis patients. J. Periodontal Res..

[129] Higashi Y., Goto C., Hidaka T., Soga J., Nakamura S., Fujii Y., Hata T., Idei N., Fujimura N., Chayama K. (2009). Oral infection-inflammatory pathway, periodontitis, is a risk factor for endothelial dysfunction in patients with coronary artery disease. Atherosclerosis.

[130] D’Aiuto F., Orlandi M., Gunsolley J.C. (2013). Evidence that periodontal treatment improves biomarkers and CVD outcomes. J. Periodontol..

[131] Ridker P.M., Morrow D.A. (2003). C-reactive protein, inflammation, and coronary risk. Cardiol. Clin..

[132] Virto L., Cano P., Jiménez-Ortega V., Fernández-Mateos P., González J., Haugen H.J., Esquifino A.I., Sanz M. (2018). Melatonin as adjunctive therapy in the treatment of periodontitis associated with obesity. J. Clin. Periodontol..

[133] Arvanitidis E., Bizzarro S., Alvarez Rodriguez E., Loos B.G., Nicu E.A. (2017). Reduced platelet hyper-reactivity and platelet-leukocyte aggregation after periodontal therapy. Thromb. J..

[134] Michelson A., Barnard M., Hechtman H., MacGregor H., Connolly R., Valeri C. (1995). Circulating degranulated platelets rapidly lose surface P-selectin but continue to circulate and function. Thrombosis and Haemostasis.

[135] Gamble J.R., Skinner M.P., Berndt M.C., Vadas M.A. (1990). Prevention of activated neutrophil adhesion to endothelium by soluble adhesion protein GMP140. Science.

[136] Marcaccini A.M., Meschiari C.A., Sorgi C.A., Saraiva M.C., de Souza A.M., Faccioli L.H., Tanus-Santos J.E., Novaes A.B., Gerlach R.F. (2009). Circulating interleukin-6 and high-sensitivity C-reactive protein decrease after periodontal therapy in otherwise healthy subjects. J. Periodontol..

[137] Priyamvara A., Dey A.K., Bandyopadhyay D., Katikineni V., Zaghlol R., Basyal B., Barssoum K., Amarin R., Bhatt D.L., Lavie C.J. (2020). Periodontal inflammation and the risk of cardiovascular disease. Curr. Atheroscler. Rep..

[138] Hopkin S., Lord J., Chimen M. (2021). Dysregulation of leukocyte trafficking in ageing: Causal factors and possible corrective therapies. Pharmacol. Res..

